# Hypericin-mediated sonodynamic therapy induces autophagy and decreases lipids in THP-1 macrophage by promoting ROS-dependent nuclear translocation of TFEB

**DOI:** 10.1038/cddis.2016.433

**Published:** 2016-12-22

**Authors:** Xuesong Li, Xin Zhang, Longbin Zheng, Jiayuan Kou, Zhaoyu Zhong, Yueqing Jiang, Wei Wang, Zengxiang Dong, Zhongni Liu, Xiaobo Han, Jing Li, Ye Tian, Yajun Zhao, Liming Yang

**Affiliations:** 1 Department of Pathophysiology, Key Laboratory of Cardiovascular Pathophysiology, Harbin Medical University, Harbin, China; 2 Department of Respiratory Medicine, The Fourth Affiliated Hospital of Harbin Medical University, Harbin, China; 3 Department of Cardiology, The First Affiliated Hospital, Cardiovascular Institute, Harbin Medical University, Harbin, China; 4 Department of Electron Microscopic Center, Basic Medical Science College, Harbin Medical University, Harbin, China; 5 Division of Cardiology, The First Affiliated Hospital, Harbin Medical University, Harbin, China

## Abstract

Lipid catabolism disorder is the primary cause of atherosclerosis. Transcription factor EB (TFEB) prevents atherosclerosis by activating macrophage autophagy to promote lipid degradation. Hypericin-mediated sonodynamic therapy (HY-SDT) has been proved non-invasively inducing THP-1-derived macrophage apoptosis; however, it is unknown whether macrophage autophagy could be triggered by HY-SDT to influence cellular lipid catabolism via regulating TFEB. Here, we report that HY-SDT resulted in the time-dependent THP-1-derived macrophage autophagy activation through AMPK/AKT/mTOR pathway. Besides, TFEB nuclear translocation in macrophage was triggered by HY-SDT to promote autophagy activation and lysosome regeneration which enhanced lipid degradation in response to atherogenic lipid stressors. Moreover, following HY-SDT, the ABCA1 expression level was increased to promote lipid efflux in macrophage, and the expression levels of CD36 and SR-A were decreased to inhibit lipid uptake, both of which were prevented by TFEB knockdown. These results indicated that TFEB nuclear translocation activated by HY-SDT was not only the key regulator of autophagy activation and lysosome regeneration in macrophage to promote lipolysis, but also had a crucial role in reverse cholesterol transporters to decrease lipid uptake and increase lipid efflux. Reactive oxygen species (ROS) were adequately generated in macrophage by HY-SDT. Further, ROS scavenger N-acetyl-l-cysteine abolished HY-SDT-induced TFEB nuclear translocation and autophagy activation, implying that ROS were the primary upstream factors responsible for these effects during HY-SDT. In summary, our data indicate that HY-SDT decreases lipid content in macrophage by promoting ROS-dependent nuclear translocation of TFEB to influence consequent autophagy activation and cholesterol transporters. Thus, HY-SDT may be beneficial for atherosclerosis via TFEB regulation to ameliorate lipid overload in atherosclerotic plaques.

Lipid catabolism disorder leads to chronic inflammation of arterial wall and subsequent atherosclerosis.^1^ Macrophages have a pivotal role in atherogenesis through regulating lipid metabolism.^2^ Normally, oxidized low-density lipoprotein (ox-LDL) is largely engulfed through scavenger receptors (SRs) of macrophage and balanced by reverse cholesterol transporters.^3, 4^ However, overloaded lipids stored in lipid droplets (LDs) impair macrophage metabolic capacity and accelerate macrophage foam cell formation, plaque rupture and clinical complications.^5, 6^ Therefore, efficient removal of lipids is essential for the prevention of foam cell formation or reverse of lipid buildup in atherosclerotic plaque and a promising strategy for the treatment of atherosclerosis.^7^

The emerging sonodynamic therapy (SDT) involving the synergistic effects of low-intensity ultrasound and a sonosensitizer was inspired by photodynamic therapy (PDT) and is characterized by dominant tissue penetration, non-invasion and regional focusing.^8^ SDT induces the generation of reactive oxygen species (ROS) and apoptosis in tumor cells, and has been shown to greatly improve the outcome of cancer patient by promoting tumor shrinkage while reducing metastases of tumor cells.^9, 10, 11, 12, 13^ We previously revealed that SDT could effectively induce apoptosis of macrophage and macrophage foam cell via mitochondrial-caspase dependent pathway^14, 15^ and rapidly stabilize atherosclerotic plaques.^16^ Meanwhile, SDT possesses high repeatability owing to its relative security and accessibility. These advance suggest that SDT could be a promising regimen against atherosclerosis.

It has been reported that natural medicine hypericin-mediated SDT (HY-SDT) induces macrophage apoptosis *in vitro*.^14^ However, the role of apoptosis in atherosclerotic lesion progression is controversial. Increased macrophage apoptosis attenuates early plaque formation, while accelerates plaque inflammation, necrosis and thrombogenicity due to defective efferocytosis in advanced atherosclerosis.^17^ Recently, numerous studies have demonstrated the importance of autophagy in cardiovascular functions via efficient efferocytosis and anti-inflammation.^18, 19, 20^ Autophagy is a highly evolutionarily conserved catabolic process constructively responsible for intracellular homeostasis, by which cytoplasmic cargo sequestered in autophagosomes undergo lysosomal degradation.^19, 21^ Clinically, increased autophagy was found in human carotid atherosclerotic plaques and associated with plaque vulnerability.^22^ In addition, increasing macrophage autophagy enhances LDs breakdown, facilitates lipid efflux and further decreases plaque susceptibility.^23, 24, 25^ In contrast, specific deficiency of macrophage autophagy exacerbates atherosclerotic lesion in high-fat-fed LDLr^−/−^ or ApoE^−/−^ mice.^2, 26^ While impaired hydrolysis of engulfed lipids results in progressive lysosomal dysfunction and accordingly autophagy defect.^27^ Therefore, manipulation of macrophage autophagy shows far-reaching therapeutic benefit for atherosclerosis. Currently, the pharmacological agents with proven autophagy modulation in atherosclerosis are as yet very few and also limited due to its adverse effects including cytokine production, dyslipidemia and hyperglycemia.^28^ Hypericin in combination with PDT has shown to induce p38(MAPK)-dependent autophagy in cancer cells and is proved a novel sonosensitizer with lower dosage and higher efficiency for SDT.^14, 29^ Whether HY-SDT could induce autophagy to overcome the adverse effects has yet not been investigated.

In this study, we investigated the occurrence of autophagy in THP-1-derived macrophage following HY-SDT and the underlying mechanism. Our results showed that HY-SDT induced AMPK/AKT/mTOR pathway dependent autophagy and decreased lipid content in macrophage through regulating ROS-dependent TFEB nuclear translocation.

## Results

### HY-SDT activates autophagy to protect macrophage from apoptosis

**Figure 1:**
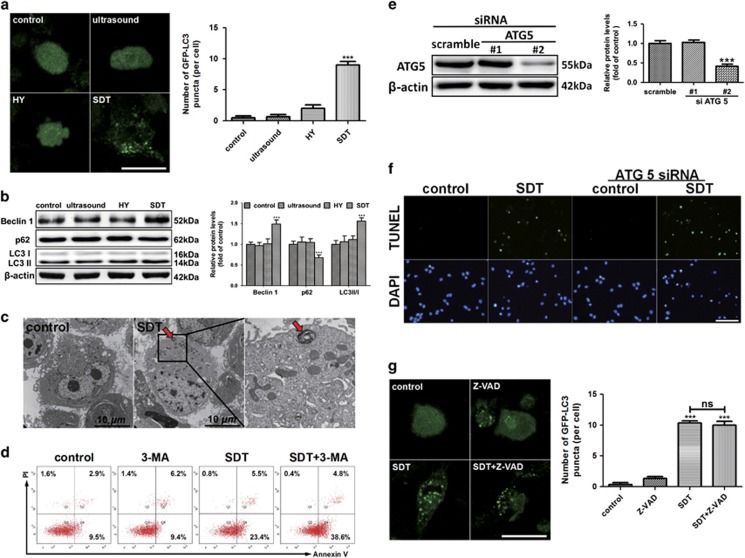
Activation of autophagy protects macrophage from HY-SDT-induced apoptosis. (**a**) Detection of autophagy in GFP-LC3 transfected macrophage treated with HY alone, ultrasound alone and HY-SDT examined under a confocal laser scanning microscope. Scale bar=20 *μ*m. (**b**) Western blot analysis of autophagy specific proteins including Beclin 1, SQSTM1/p62 and LC3 II/I in macrophage at 6 h after different treatments as indicated. (**c**) TEM analysis of morphological change of HY-SDT-treated macrophage at 6 h, showing a typical autolysosome (myelin figures) in the HY-SDT group (red arrow). (**d**) Macrophage treated with HY-SDT in the presence or absence of 10 mM 3-MA were stained with Annexin V-FITC/PI, followed by flow cytometry analysis of the effect of HY-SDT-induced autophagy on apoptosis. (**e**) Representative western blots of ATG5 following siRNA treatment. (**f**) TUNEL staining in HY-SDT-treated macrophage in the presence of ATG5 siRNA. Scale bar=100 *μ*m. (**g**) Detection of the effect of apoptosis inhibitor Z-VAD on the autophagy activation following HY-SDT. The GFP-LC3 transfected macrophage treated with or without Z-VAD were examined under a confocal laser scanning microscope. Scale bar=20 *μ*m. ***P<0.001 *versus* control; NS, no significance. All values are given as mean±S.D. (error bars) of three independent experiments

Previous studies have indicated that macrophage autophagy has a protective role in the pathogenesis of atherosclerosis,^2, 30^ and autophagy shares many common stimuli with apoptosis.^31, 32^ To explore the role of HY-SDT on autophagy, we assessed macrophage autophagy by GFP-LC3 transfection^32^ and acridine orange staining^33^ at 6 h following HY-SDT. The results showed that autophagy was activated in the HY-SDT group as evidenced by a substantial increase in the number of GFP-LC3 puncta (Figure 1a) as well as the puncta of red fluorescence by acridine orange staining compared with either treatment alone (Supplementary Figure S1). Meanwhile, HY-SDT increased the conversion of LC3 I to LC3 II (LC3 II/I) and Beclin 1 expression which are specific autophagy markers,^34^ while decreased SQSTM1/p62^26^ (Figure 1b). The activation of autophagy was further illustrated by transmission electron microscopy (TEM),^35^ showing myelin figures (autolysosomes) typical of autophagy in cells following HY-SDT (Figure 1c). These data demonstrate that HY-SDT induces autophagy in macrophage.

The crosstalk between autophagy and apoptosis is complex, either antagonizing or synergizing.^30, 36^ We previously reported that HY-SDT induced apoptosis in macrophage.^14^ To explore the relationship between autophagy and apoptosis that are induced by HY-SDT, we detected the apoptosis of macrophage using Annexin V/PI and TUNEL assay in the presence of autophagy inhibitor 3-methyladenine (3-MA) or autophagy specific deficiency via ATG5 knockdown.^37^ Autophagy suppression by 3-MA significantly augmented the apoptosis induced by HY-SDT (Figure 1d). We next knocked down ATG5 by siRNAs and found ATG5 siRNA #2 decreased the protein level of ATG5 (Figure 1e). Macrophages were subjected to HY-SDT with or without ATG5 siRNA #2, and cell apoptosis was assessed by TUNEL staining. As shown in Figure 1f, downregulation of ATG5 significantly enhanced HY-SDT-induced apoptosis. However, there was no statistically significant difference in the number of fluorescent puncta of cells between HY-SDT (10.3±0.6) and HY-SDT+Z-VAD (10.0±1.0) groups transfected by GFP-LC3 (Figure 1g), and the results corresponded well with monodansylcadaverine staining (Supplementary Figure S2). Collectively, autophagy is activated in macrophage following HY-SDT and displays antiapoptotic effect.

### HY-SDT-induced autophagy occurs earlier than apoptosis in macrophage

**Figure 2:**
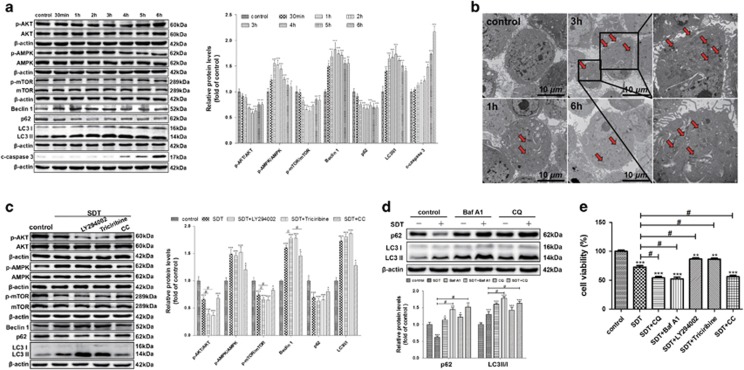
HY-SDT-induced macrophage autophagy occurs earlier than apoptosis and through AMPK/AKT/mTOR pathway. (**a**) Western blot analysis of autophagy signaling pathway proteins at the indicated time following HY-SDT. (**b**) TEM analysis of autophagy activation in macrophages at the indicated time following HY-SDT (red arrow, autolysosome). (**c**) Western blot analysis of the major involvement of autophagy signaling pathway during HY-SDT in the presence or absence of different inhibitors. LY294002, Triciribine and CC are PI3K, AKT and AMPK inhibitors, respectively. (**d**) Western blot analysis of autophagic flux through SQSTM1/p62 and LC3 II/I in the presence of autophagic flux inhibitors, including Baf A1 (vacuolar-type H^+^-ATPase inhibitor) and CQ. (**e**) The effect of different autophagy inhibitors on cell viability following HY-SDT measured by CCK-8 assay. *P<0.05, **P<0.01, ***P<0.001 *versus* control, ^#^*P*<0.05 *versus* SDT. All values are given as mean±S.D. (error bars) of three independent experiments

Autophagy and apoptosis have been reported to be activated at different time points following SDT treatment.^32^ To explore the dynamic activation of autophagy, we detected the expression levels of Beclin 1, SQSTM1/p62 and LC3 II/I at different time points following HY-SDT. As shown in Figure 2a, all these proteins were altered in a time-dependent manner following HY-SDT. Beclin 1 and LC3 II/I increased and peaked at 2 h and 3 h and SQSTM1/p62 decreased to the lowest level at 3 h. Consisting with the protein levels, ultrastructural analysis by TEM showed numerous autolysosomes at 3 h (Figure 2b) as well as increased GFP-LC3 puncta formation (Supplementary Figure S3). Meanwhile, we detected the expression level of cleaved caspase 3 (c-caspase 3) that is a critical executioner of apoptosis. We found the cleaved caspase 3 increased from 4 h and peaked at 6 h following HY-SDT (Figure 2a). These results indicate that HY-SDT-induced autophagy peaks at 3 h post treatment, which is earlier than apoptosis at 6 h.^14^

### HY-SDT induces autophagy through AMPK/mTOR and AKT/mTOR pathways in macrophage

AKT/mTOR and AMPK/mTOR signaling cascades have a key role in regulating autophagy.^24, 38^ To explore the molecular mechanism underlying HY-SDT-induced autophagy, we additionally analyzed the phosphorylation levels of AKT, AMPK and mTOR (p-AKT, p-AMPK and p-mTOR) at different time points following HY-SDT. As show in Figure 2a, HY-SDT induced a rapid and significant increase in p-AMPK from 1 h to 3 h, while a reduction of p-AKT with the minimum at 3 h, suggesting the involvement of AKT and AMPK in HY-SDT-induced autophagy. To further support this observation, the cells of HY-SDT were treated with or without of selective inhibitors of class I PI3K (LY294002), AKT (Triciribine) or AMPK (Compound C, CC).^39, 40^ LY294002 and Triciribine suppressed the phosphorylation of AKT and mTOR, whereas CC inhibited the phosphorylation of AMPK. Consistently, LY294002 and Triciribine further increased the expression levels of LC3 II/I and Beclin 1, while decreased SQSTM1/p62 expression level following HY-SDT. In contrast, CC prevented HY-SDT-induced increase of LC3 II/I and Beclin 1 (Figure 2c). Collectively, these data provide a strong evidence of AMPK/mTOR-dependent induction of autophagy by HY-SDT.

At the final stage of autophagy, autophagosome fuses with lysosome to degrade autophagosomal contents including LC3 II and SQSTM1/p62 by lysosomal hydrolase, which is named autophagic flux.^34^ To more exactly examine HY-SDT-induced autophagy, macrophages were pre-treated with bafilomycin A1 (Baf A1) or chloroquine (CQ), which are lysosomal inhibitors preventing the fusion and subsequent proteolysis. Compared with HY-SDT alone, HY-SDT in combination with either Baf A1 or CQ further increased the expression levels of LC3 II/I and SQSTM1/p62, suggesting that HY-SDT promoted the formation of both autophagosome and autophagic flux through lysosomes (Figure 2d). In addition, Baf A1, CQ and CC significantly enhanced while LY294002 and Triciribine prevented the cell death induced by HY-SDT (Figure 2e). Altogether, the AMPK/mTOR pathway dependent autophagy activation protects macrophage from HY-SDT-mediated cell death requiring the full process of autophagic flux through lysosomes.

### ROS contribute to HY-SDT-induced autophagy in macrophage

**Figure 3:**
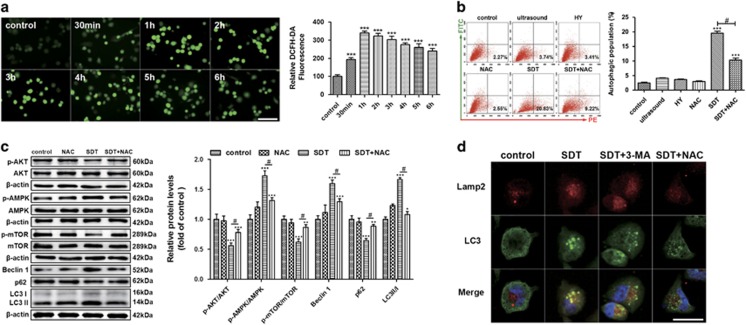
The effect of ROS on the initiation of HY-SDT-induced macrophage autophagy. (**a**) Fluorescence microscopy analysis of fluorescence intensity of ROS in macrophage at indicated time following HY-SDT loading with DCFH-DA. Scale bar=100 *μ*m. (**b**) Flow cytometry analysis of the effect of ROS on the initiation of HY-SDT-induced macrophage autophagy in the presence of NAC as stained by acridine orange at 3 h following HY-SDT. (**c**) Western blot analysis of ROS effect on autophagy signaling pathway-related proteins in macrophage at 3 h following HY-SDT. (**d**) Immunofluorescence analysis of ROS effect on autolysosome formation via detecting the co-localization between LC3 (showing autophagosome) and Lamp2 (showing lysosome) at 3 h following HY-SDT in macrophage. Scale bar=20 *μ*m. *P<0.05, **P<0.01, ***P<0.001 *versus* control, ^#^*P*<0.05 *versus* SDT. All values are given as mean±S.D. (error bars) of three independent experiments

It has been shown that ROS are closely related to activation of apoptosis as well as autophagy in response to SDT in cancer cells.^32^ Here, HY-SDT resulted in the production of ROS as early as 30 min with a further extension of the effect up to 1 h (Figure 3a). We next assessed whether ROS are involved in HY-SDT-induced autophagy. We found that ROS scavenger N-acetyl-l-cysteine (NAC) treatment led to a significant reduction in the accumulation of acidic vesicular organelles (Figure 3b), and reverse of the changes of all the proteins involved in AMPK/AKT/mTOR pathway and autophagy at 3 h (Figure 3c). In addition, the increase of fluorescent puncta in the HY-SDT group via monodansylcadaverine staining were prevented by 3-MA or NAC (Supplementary Figure S4). To assess the role of ROS in HY-SDT-induced autophagic flux, we analyzed the formation of autolysosome by double immunofluorescence staining. As shown in Figure 3d, the green LC3 dots (indication of autophagosome) largely overlapped with red Lamp2 (indication of functional lysosome) in the HY-SDT group thereby showing yellow fluorescence, while was abolished by 3-MA or NAC. These results demonstrate that the induction of ROS contribute to the AMPK/mTOR pathway dependent macrophage autophagy activation following HY-SDT.

### HY-SDT stimulates ROS-dependent TFEB nuclear translocation in macrophage

**Figure 4:**
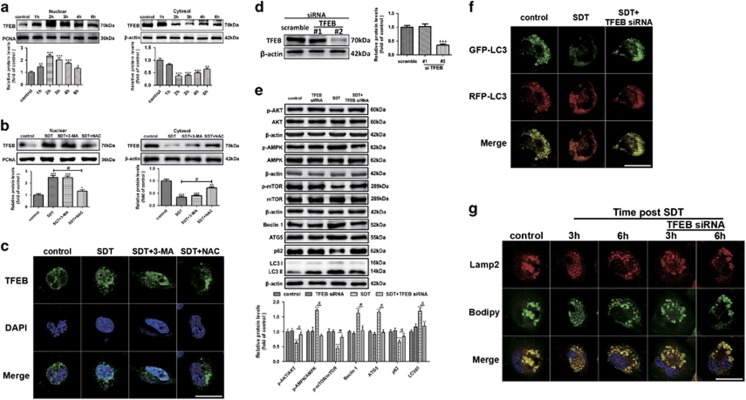
ROS-dependent TFEB nuclear translocation activates autophagy, promoted lysosome regeneration and enhanced lipid catabolism in macrophage following HY-SDT. (**a**) Western blot analysis of subcellular distribution of TFEB in macrophage between nucleus and cytoplasm at specific time following HY-SDT. (**b** and **c**) Western blot and immunofluorescence analysis of the effects of 3-MA and NAC on TFEB translocation in macrophage at 2 h following HY-SDT. Scale bar=20 *μ*m. (**d**) Representative western blots of TFEB following siRNA treatment. (**e**) Western blot analysis of TFEB effect on the activation of macrophage autophagy signaling pathway-related proteins. (**f**) Immunofluorescence analysis of autophagy activation in macrophage transfected with Lentivirus expressing LC3 in the presence of TFEB siRNA at 3 h following HY-SDT. Scale bar=20 *μ*m. **(g)** Immunofluorescence analysis of TFEB effect on lysosomal dysfunction in macrophage as assessed Bodipy (green) and Lamp2 (red) staining at 3 h and 6 h following HY-SDT. Scale bar=20 *μ*m. *P<0.05, **P<0.01, ***P<0.001 *versus* control, ^#^*P*<0.05 *versus* SDT. All values are given as mean±S.D. (error bars) of three independent experiments

Transcription factor EB (TFEB) is a recently discovered key regulator of autophagy-lysosome pathway to promote protein clearance.^25, 41^ TFEB could upregulate the expression of nearly two-thirds of autophagy-lysosome genes and its overexpression shows vast therapeutic effects in cardiovascular disease by rescuing lipid-induced lysosomal dysfunction and downstream sequelae,^25, 42, 43^ as well as enhancing lipolysis.^44^ Normally, TFEB is located in the cytosol and on lysosomal surface where interacting with mTOR in an inactive phosphorylated form,^45^ while translocating to nucleus in response to stimuli.^24^ To explore the potential role of TFEB in HY-SDT-induced autophagy, we assessed the intracellular localization of TFEB following HY-SDT. In cell fractionation experiments, we found that HY-SDT resulted the nuclear translocation and accumulation while decrease of TFEB in the cytosol mostly at 2 h, which was gradually recovered from 3 h to 6 h (Figure 4a). In addition, HY-SDT led to the gradual dissociation of TFEB from mTOR (Supplementary Figure S5). To discriminate the role of ROS generation and autophagy activation in TFEB nuclear translocation in cells following HY-SDT, we tested the effects of ROS scavenger NAC and autophagy inhibitor 3-MA on TFEB nuclear translocation. Here, we found that NAC but not 3-MA inhibited TFEB nuclear translocation as demonstrated by western blot in cell fractions and immunofluorescence analysis of TFEB. The expression levels of TFEB in the HY-SDT group with or without of 3-MA had no statistically significant difference neither in the nuclear fractionation, nor in the cytosol fractionation (Figure 4b). Similarly, the immunofluorescence of TFEB co-localized well with the nucleus in the HY-SDT group with or without of 3-MA (Figure 4c). Although all these changes of western blot and immunofluorescence in cells following HY-SDT were reversed in the presence of NAC, suggesting that the nuclear translocation of TFEB triggered by HY-SDT is ROS-dependent and autophagy might be the downstream effector of TFEB. To this hypothesis, we knocked down TFEB by siRNAs and found TFEB siRNA #2 decreased the protein level of TFEB (Figure 4d). Macrophages were then subjected to HY-SDT with or without TFEB siRNA #2, and cell autophagy-related proteins were assessed by western blot analysis. The results revealed that TFEB siRNA blocked the changes of autophagy-related proteins following HY-SDT (Figure 4e). To further assess the effect of TFEB on autophagic flux, we transfected cells with mRFP-GFP-LC3 (red fluorescent protein–green fluorescent protein).^34^ HY-SDT group showed more red fluorescence compared with the bright yellow fluorescence in the control group, which was prevented by TFEB siRNA (Figure 4f). These data clearly indicate that ROS-dependent TFEB nuclear translocation is the upstream factor of autophagy activation following HY-SDT.

### HY-SDT promotes TFEB-dependent lysosome regeneration in macrophage

The lipid-rich LDs are degraded by lysosome via autophagy,^46^ which is seriously impaired in advanced atherosclerosis.^27^ To investigate the influence of HY-SDT on lysosome functions, ox-LDL activated macrophages were double-fluorescent labeled with Lamp2 antibody and Bodipy. Confocal microscopy images showed that lysosomes became larger, a feature of lysosomal storage disorders, and co-localized with many Bodipy^+^ LDs in the control group.^25, 47^ In contrast, Bodipy^+^ LDs co-localized with more and smaller lysosomes, a feature of new reformed functional lysosome,^48^ in the HY-SDT group at 3 h and markedly decreased at 6 h. All of these changes were reversed by TFEB siRNA (Figure 4g). These results indicate that HY-SDT promotes lysosome regeneration in a TFEB-dependent manner to improve lipolysis.

### HY-SDT enhances lipid catabolism of macrophage via regulating ROS-dependent TFEB nuclear translocation

**Figure 5:**
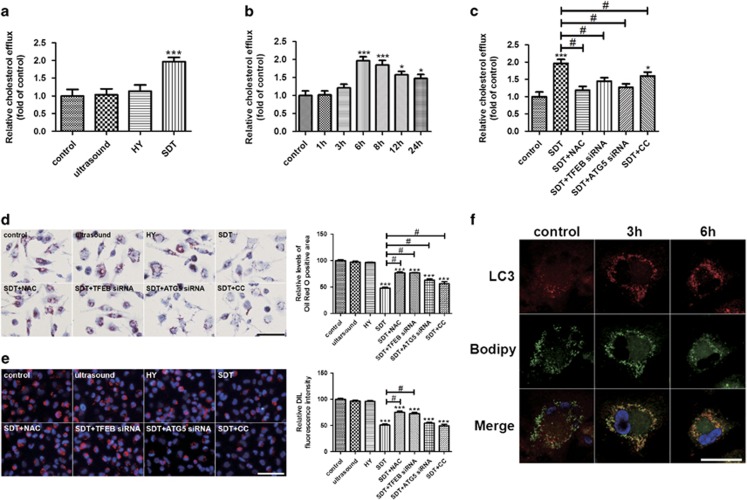
HY-SDT enhances the lipid catabolism of macrophage via regulating ROS-dependent TFEB nuclear translocation. (**a**) Cholesterol efflux to HDL (50 *μ*g/well) in macrophage at 6 h after different treatments. (**b**) Cholesterol efflux to HDL (50 *μ*g/well) in macrophage for the indicated time following HY-SDT. (**c**) Cholesterol efflux to HDL (50 *μ*g/well) in macrophage pre-treatment with NAC, TFEB siRNA, ATG5 siRNA and CC at 6 h following HY-SDT. (**d**) Lipids accumulation in macrophage pre-treatment with NAC, TFEB siRNA, ATG5 siRNA and CC was determined by ORO staining. Scale bar=50 *μ*m. (**e**) Fluorescence microscopy analysis of the uptake of DIL-ox-LDL in macrophage pre-treatment with NAC, TFEB siRNA, ATG5 siRNA and CC at 6 h following HY-SDT. Scale bar=100 *μ*m. **(f)** Immunofluorescence analysis of lipid catabolism through double fluorescence labeling with Bodipy and anti-LC3 at 3 h and 6 h following HY-SDT. Scale bar=20 *μ*m. *P<0.05, **P<0.01, ***P<0.001 *versus* control, ^#^*P*<0.05 *versus* SDT. All values are given as mean±S.D. (error bars) of three independent experiments

To assess whether HY-SDT-induced autophagy has the similar effect on lipid degradation as previous study,^46^ the lipid efflux following HY-SDT was evaluated by fluorescently labeled cholesterol. HY-SDT effectively promoted macrophage cholesterol efflux mostly at 6 h (Figures 5a and b) and reduced the cellular lipids accumulation as stained by Oil red O (ORO), both of which were reversed by NAC, TFEB siRNA, ATG5 siRNA and CC (Figures 5c and d). These results suggest that the increased lipid efflux following HY-SDT is related to ROS-dependent TFEB nuclear translocation and subsequent AMPK-dependent autophagy activation. We then explored whether the lipid content reduction was also related to the reduced uptake. 1,1'-dioctadecyl-3,3,3',3'-tetramethylindocarbocyanine perchlorate labeled ox-LDL (DIL-ox-LDL) binding assay showed HY-SDT-decreased lipid uptake of macrophage, which was reversed by NAC and TFEB siRNA but not ATG5 siRNA and CC (Figure 5e), indicating that the decreased lipid uptake is related to ROS and TFEB. To visually observe the breakdown of lipids in LDs, we detected the co-localization between autophagosomes and LDs. Consistent with previous finding,^49^ HY-SDT led to an increased number of LC3^+^ autophagosomes along with a decreased number of Bodipy^+^ LDs at 6 h (Figure 5f). These results demonstrate that HY-SDT decreases lipid uptake, enhances lipid breakdown and efflux through autophagy activation via ROS-dependent TFEB nuclear translocation.

### HY-SDT increases ABCA1-dependent lipid efflux and decreases CD36 and SR-A-mediated lipid uptake

**Figure 6:**
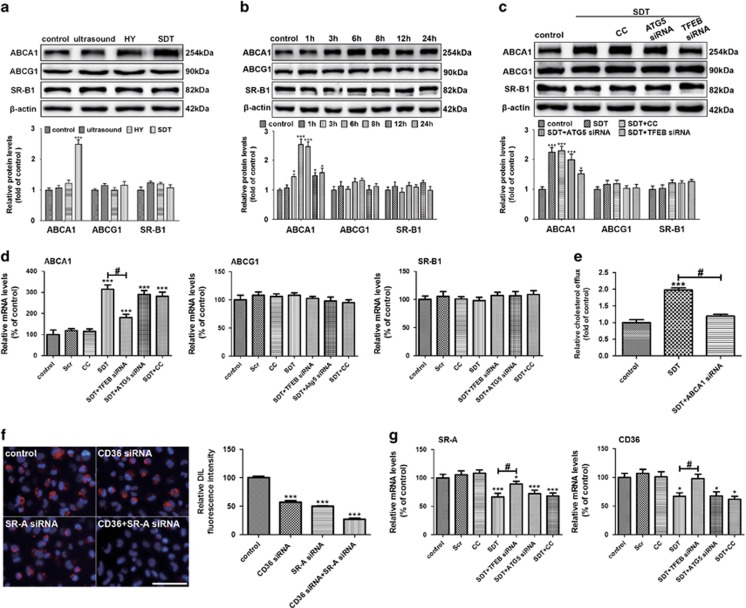
HY-SDT promotes the lipid efflux through ABCA1 and inhibits lipid uptake by downregulating CD36 and SR-A in macrophage. (**a**) Western blot analysis of cholesterol efflux transporters ABCA1, ABCG1 and SR-B1 of macrophage at 6 h after different treatments. (**b**) Western blot analysis of cholesterol efflux transporters of macrophage at indicated time following HY-SDT. (**c**) Western blot analysis of cholesterol efflux transporters of macrophage pre-treatment with CC, ATG5 siRNA and TFEB siRNA at 6 h following HY-SDT. (**d**) RT-PCR analysis of mRNA expression levels of cholesterol efflux transporters in macrophage pre-treatment with CC, ATG5 siRNA and TFEB siRNA at 6 h following HY-SDT. (**e**) Cholesterol efflux to HDL (50 *μ*g/well) in macrophage at 6 h following HY-SDT in the presence of ABCA1 siRNA. (**f**) Fluorescence microscopy analysis of the uptake of DIL-ox-LDL in macrophage in the presence of CD36 siRNA and SR-A siRNA. Scale bar=100 *μ*m. (**g**) RT-PCR analysis of mRNA expression levels of cholesterol uptake transporters pre-treatment with CC, ATG5 siRNA and TFEB siRNA at 6 h following HY-SDT. *P<0.05, ***P<0.001 *versus* control, ^#^*P*<0.05 *versus* SDT. All values are given as mean±S.D. (error bars) of three independent experiments

Since lipid efflux is closely related to reverse cholesterol transporters, including SR-B1, ATP-binding membrane cassette transport protein A1 (ABCA1) and G1 (ABCG1),^50^ we further investigated whether these transporters were involved in the increased lipid efflux of macrophage induced by HY-SDT. As shown in Figure 6a, HY-SDT (2.5±0.3) significantly increased the expression level of ABCA1 compared with the control group (1.0±0.1), whereas it had no statistically significant effect on the expression levels of ABCG1 (1.2±0.2) or SR-B1 (1.1±0.2) compared with the control group (1.0±0.1 and 1.0±0.2 respectively). Following HY-SDT, the protein levels of ABCA1 increased in a time-dependent manner peaking at 6 h (Figure 6b), which was significantly blocked by TFEB siRNA other than ATG5 siRNA or CC (Figure 6c), corresponding well with the mRNA levels (Figure 6d). To further support the critical role of ABCA1 expression in the increased lipid efflux of macrophage following HY-SDT, we detected the lipid efflux of macrophage following HY-SDT with or without ABCA1 knockdown via ABCA1 siRNA. We found ABCA1 siRNA #2 decreased the protein level of ABCA1 (Supplementary Figure S6a). Moreover, the lipid efflux promoted following HY-SDT was reversed by ABCA1 siRNA (Figure 6e). These results suggest that ABCA1 is mainly responsible for the lipid efflux and the increased ABCA1 expression is related to TFEB nuclear translocation other than autophagy following HY-SDT. Class A SR (SR-A) and class B SR (CD36) have been demonstrated the primary receptors for lipid uptake of macrophage.^3^ To explore the relationship among CD36, SR-A and lipid uptake of macrophage, we detected the lipid uptake capacity of macrophage by DIL-ox-LDL binding assay following knockdown of CD36 and SR-A via siRNAs (Supplementary Figures S6b and c). The results showed that the uptake of DIL-ox-LDL decreased significantly in the double genes knockdown group (Figure 6f), implying that CD36 and SR-A are the primary receptors responsible for lipid uptake of macrophage. Moreover, the mRNA levels of SR-A and CD36 decreased significantly by HY-SDT, which were reversed by TFEB siRNA (Figure 6g). All these data indicate that HY-SDT promotes lipid catabolism mainly through increasing ABCA1-dependent lipid efflux and decreasing CD36 and SR-A-mediated lipid uptake via ROS-dependent TFEB nuclear translocation.

## Discussion

**Figure 7:**
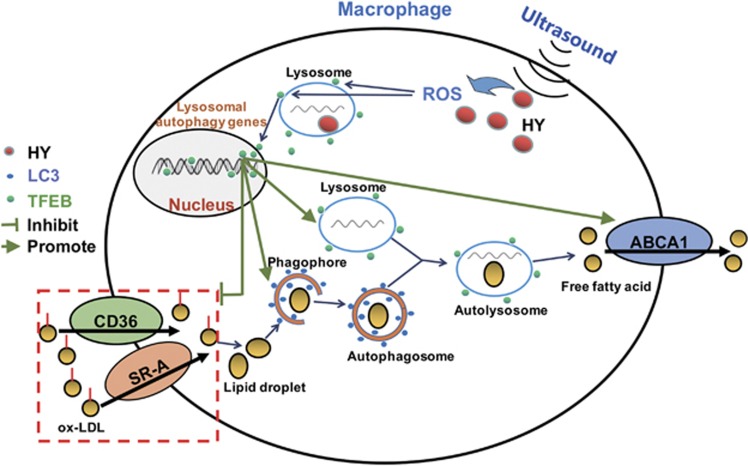
Proposed model describing the mechanism of autophagy activation and lipid catabolism by HY-SDT. ROS generated by ultrasound-activated HY are the key regulator for TFEB translocation from lysosome to nucleus to bind corresponding DNA sequences, which triggers autophagy activation and lysosome regeneration. Then the LDs derived from the engulfed ox-LDL via CD36 and SR-A are degraded to free fatty acid by autophagy activation. Moreover, TFEB nuclear translocation enhances ABCA1 expression to promote free fatty acid efflux, meanwhile decreases the expression levels of CD36 and SR-A to inhibit lipid uptake. Therefore, HY-SDT displays an efficient treatment for decreasing the lipid content of macrophage

In this study, we provided the first evidence showing that ROS-dependent TFEB nuclear translocation was involved in the trigger of macrophage autophagy, moreover, ameliorated lysosome damage and reduced intracellular lipid content following HY-SDT. Our findings support a model of the possible mechanism of autophagy activation by HY-SDT and its effect on macrophage (Figure 7), suggesting that HY-SDT is a potential therapeutic avenue for atherosclerosis.

Autophagy and apoptosis can be activated by many common stimuli.^31^ Our results showed that HY-SDT triggered autophagy earlier than apoptosis via mTOR suppression. Moreover, p-AKT mediating lipid efflux and plaque stabilization via regulating mTOR was downregulated by HY-SDT.^39, 51^ However, PI3K/AKT signaling inhibitors markedly increased LC3 II/I in the HY-SDT group, indicating some other pathways are also involved in the autophagy regulation. Notably, AMPK is not only an important intracellular energy sensor, but also a potential target of drugs for cardiovascular diseases via regulating autophagy through mTOR.^52^ Here we found HY-SDT increased p-AMPK along with LC3 II/I, which were particularly inhibited by AMPK inhibitor other than PI3K and AKT inhibitors. Altogether, macrophage autophagy was activated following HY-SDT by mTOR suppression via regulating both AMPK and AKT, and AMPK is the main upstream factor.

An intricate crosstalk has been reported to exist between autophagy and apoptosis.^31^ Some researches show that autophagy has a protective role in human vascular smooth muscle cell and macrophage against apoptosis induced by atherogenic lipids.^30, 50^ Although other studies indicate that autophagy promotes the elimination of damaged cells by apoptosis in cancer cells.^36^ In this study, the results showed autophagy inhibition increased apoptosis following HY-SDT implying that HY-SDT-induced autophagy has antiapoptotic effect. The key components of apoptotic machinery also prompt autophagy through molecular interactions with autophagy proteins.^53^ Beclin 1 is the major regulator node between them by directly binding Bcl-2 to suppress autophagy,^54^ whereas it dissociates from Bcl-2 to activate autophagy when phosphorylated by AMPK.^55^ Consistently, HY-SDT induced the time-dependent increase of autophagy as well as p-AMPK and Beclin 1 reversed by AMPK inhibitor CC. Meanwhile, the released Beclin 1 is also reported to trigger apoptosis when translocating to the outer mitochondrial membrane. Therefore, the role of Beclin 1 in autophagy and apoptosis induced by HY-SDT needs further investigation.

ROS as natural byproducts of metabolism have important roles in homeostasis maintenance.^56^ Further, some studies report that ROS are the primary effectors for SDT efficiency by regulating multiple signaling pathways.^32^ In the present study, a rapid generation of ROS was caused at 30 min and peaked at 1 h following HY-SDT. However, it has been reported that ROS has short movement distance and half-time. This discrepancy is probably due to the initial slightly increased ROS triggers a mitochondrial burst of ROS generation, called 'ROS-induced ROS release'.^57^ In addition, the elimination of ROS with NAC markedly relieved HY-SDT-elicited autophagy and the changes of AMPK/AKT/mTOR pathway proteins, suggesting that ROS generation is a relatively earlier event than AMPK activation. These data support the upstream role of ROS accumulation leading to HY-SDT-induced autophagy via AMPK phosphorylation that inhibits mTOR.^58^

During atherosclerosis, lipid overload in lysosomes leads to progressive dysfunction of lysosome and autophagy.^25, 59^ Our results showed that ROS-dependent TFEB nuclear translocation had a crucial role in activating autophagy, promoting lysosome regeneration and reducing lipid content of macrophage stimulated by ox-LDL following HY-SDT. First, ROS inhibition blocked TFEB nuclear translocation and silencing TFEB completely reversed autophagy-related proteins activation as well as the formation of autolysosome. Second, dysfunctional lysosomes in atherosclerotic macrophage usually show engorgement and diminished proteolytic capacity, which is rescued by TFEB via initiating the biogenesis of lysosome and numerous lysosomal enzymes.^25, 59^ In this study, the larger lysosomes stimulated by ox-LDL became smaller as well as Bodipy^+^ LDs at 6 h following HY-SDT, while restored to the aberrant status when TFEB was silenced by siRNA, indicating that lysosome biogenesis was triggered via TFEB regulation following HY-SDT to enhance the overall degradative capacity of cells. Third, efficient lipid catabolism is atheroprotective, whereas aberrant accumulation of lipid would exacerbate the inflammatory milieu and plaque progression.^25, 60^ Macrophage autophagy responsible for hydrolyzing LDs into free fatty acids is a crucial nexus for lipid efflux in atherosclerosis.^60, 61^ Our results showed the increased co-localization of autophagosome and lysosome correlated with decreased LDs. Moreover, from fluorescently labeled cholesterol assay and ORO assay, the increased lipid efflux and decreased lipid accumulation by HY-SDT were reversed by NAC, silence of TFEB and ATG5, and CC, suggesting that ROS-dependent TFEB nuclear translocation has a key role in HY-SDT promoting lipid breakdown and efflux of macrophage. Fourth, cholesterol transporters are closely related to cholesterol metabolism.^3, 49^ In this study, cholesterol transporter ABCA1 was increased in a time-dependent manner at both transcriptional and translational levels following HY-SDT. Simultaneously, the increased lipid efflux was significantly reversed by ABCA1 silence indicating that ABCA1 is the primary transporter responsible for the increased lipid efflux following HY-SDT. Furthermore, the increases of both lipid efflux and ABCA1 expression were inhibited by TFEB silence, suggesting the pivotal role of TFEB in regulating cholesterol transporters. Besides, the decreased lipid uptake capacity of macrophage as well as mRNA levels of CD36 and SR-A (the primary receptors for lipid uptake of macrophage) following HY-SDT were all reversed by TFEB, suggesting that HY-SDT reduces lipid uptake of macrophage via downregulation of CD36 and SR-A. All these results indicated that lipid catabolism was regulated by ROS-dependent TFEB nuclear translocation following HY-SDT.

Taken together, our findings demonstrated the novel efficiency of HY-SDT on macrophage by increasing macrophage autophagy to facilitate lipolysis and reduce lipid content via regulating TFEB. Thus, HY-SDT could be a promising regimen against atherosclerosis.

## Materials and Methods

### Reagents

NAC (1 mM), 3-MA (10 mM), Baf A1 (25 nM), acridine orange (5 *μ*g/ml) and monodansylcadaverine (20 mM) were purchased from Sigma Chemical Co. (St. Louis, MO, USA). LY294002 (10 *μ*M), Triciribine (20 *μ*M), CC (10 *μ*M) and CQ (20 *μ*M) were purchased from Selleckchem (Houston, TX, USA). Antibodies against phospho-mTOR/mTOR, phospho-AKT/AKT, phospho-AMPK/AMPK, LC3, ATG5 and TFEB were purchased from Cell Signaling Technology (Beverly, MA, USA); SQSTM1/p62, ABCA1 and Beclin 1 were from Abcam (Cambridge, UK); ABCG1 and SR-B1 are from NOVUS biologicals (LLC, USA); Lamp2 was from Santa Cruz Biotechnology (Santa Cruz, CA, USA); *β*-actin and horseradish peroxidase-conjugated secondary antibodies were from ZSGB-BIO (Beijing, China). All inhibitors were loaded with cells 1 h before ultrasonic treatment. The inhibitors did not display any cytotoxicity to the cells.

### Cell culture, sonodynamic therapy protocol

Human THP-1 monocytes were purchased from American Type Culture Collection (Manassas, VA, USA) and cultured as previously described.^14^ For experiments, the cells at logarithmic growth phase were seeded at 1.0 × 10^5^ cells/ml in 100 ng/ml phorbol-12-myristate-13-acetate (PMA, La Jolla, CA, USA) for 72 h and differentiated into adherent THP-1 derived macrophages. To avert the activation of autophagy by starvation, all of the studies were carried on in complete medium. The sonication device used here and condition of HY-SDT (0.25 *μ*g/ml HY, 0.4 W/cm^2^, 15 min ultrasound exposure time) was as the same as previously described.^15^

### Transmission electron microscopy

Conventional TEM is the gold standard for autophagy diagnosis according to the formation of autophagosome and autolysosome. At specific times following different treatments, macrophages were harvested by trypsinization and detected as previously described.^14^ The images of ultra thin slices were observed and obtained in TEM (JEM-1220, JEOL, Tokyo, Japan).

### Annexin V/PI flow cytometric assay

Annexin V-FITC/propidium iodide (PI) Apoptosis Detection Kit (BD Pharmingen, Franklin Lakes, NJ, USA) was used to detect apoptosis according to the manufacturer's instructions. Six hours following HY-SDT with or without 3-MA, the cells were collected and incubated with 5 *μ*l Annexin V-FITC and 10 *μ*l PI for 15 min at room temperature in the dark. After filtration, the cells were analyzed by fluorescence-activated cell sorting Calibur system within 1 h. Annexin V^+^/PI^-^ and Annexin V^+^/PI^+^ represent the apoptotic cells in early and late phase, respectively. The data were analyzed using BD GACSDiva Software v7.0 (Becton-Dickinson, USA).

### Transfection

The knockdown of ATG5 or TFEB in macrophage was obtained by siRNAs. An irrelevant 21-nucleotide siRNA was used as a negative control (GenePharma, Shanghai, China). The target sequences were as follows: ATG5-sh#1: 5′-CCAUCAAUCGGAAACUCAUTT-3′, ATG5-sh#2: 5′-GCAGUGGCUGAGUGAACAUTT-3′, TFEB-sh#1: 5′-CCGAGACCUAUGGGAACAATT-3′, TFEB-sh#2: 5′-CAGGCUGUCAUGCAUUACATT-3′, SR-A-sh#1: 5′-GGAGCGUGUUUACAAUGUATT-3′, SR-A-sh#2: 5′-GGGAAUGCAAUAGAUGAAATT-3′, CD36-sh#1: 5′-CCAUCUUCGAACCUUCACUTT-3′, CD36-sh#2: GUUGCCAUAAUCGACACAUTT, ABCA1-sh#1: 5′-GGCCUCUAUUUAUCUUCCUTT-3′, ABCA1-sh#2: 5′- CCCAUCAUGUCAAGUACAATT-3′. The efficiency of transfection was confirmed using western blotting analysis.

The macrophages were transfected with lentivirus and adenovirus vectors encoding LC3 (pGMLV-CMV-RPF-GFP-LC3/GFP-LC3, GenePharma). The transfection efficiency was detected by confocal laser scanning microscope LSM 510 Meta; Zeiss, Gottingen, Germany) according to the percentage of fluorescence positive cells (>80%). The co-localized yellow fluorescence of both GFP and RFP represents autophagosomes. In autolysosomes, it shows less co-localization and stronger red fluorescence because of quenching of GFP signal in acidic compartments. In GFP-LC3 transfected cells, punctate pattern dots represent LC3 activation.

### Terminal dUTP nick end-labeling (TUNEL) assay

HY-SDT-induced apoptosis preconditioned with ATG5 siRNA interference was detected by the *In Situ* Cell Death Detection Kit, POD (Roche, Mannheim, Germany) according to the manufacturer's instructions. After TUNEL reaction, nuclei were counterstained with 1.0 *μ*g/ml DAPI (Roche, Mannheim, Germany) for 5 min to highlight nucleus and observed under a fluorescence microscope (Olympus IX81, Japan). Only TUNEL-positive cells that co-localize with DAPI-stained nuclei were counted as positive.

### CCK-8 assay

The cell viability was assessed by the cell counting kit 8 (CCK-8) assay (Beyotime, Beijing, China) as described previously.^14^ Live cells were counted according to the optical density (OD) of each well which was quantified by an enzyme-linked immunosorbent assay microplate reader (Varian Australia Pty Ltd, Melbourne, VIC, Australia) at 450 nm. The OD of the results was indicated as the percentage of cell viability in the control group that was set as 100%.

### Immunofluorescence assay

After treatments, cells seeded in glass-bottom cell culture dishes were fixed in 4% paraformaldehyde and permeabilized with 0.1% Triton X-100 followed by blocking with 3% bovine serum albumin (BSA). Then, the cells were immunolabelled with specific primary antibodies (TFEB, LC3 and Lamp2 at 1:100 ratio) overnight at 4 °C. After rinsing, the cells were incubated with corresponding TRITC- or FITC-conjugated secondary antibody (1:250) in 1% BSA or Bodipy (Invitrogen) for neutral lipid staining for 1 h at 37 °C. Nuclei were stained with DAPI for 5 min at room temperature. The fluorescence of cells was examined by confocal laser scanning microscope.

### ROS detection

The ROS generation in cells after HY-SDT was evaluated according to the fluorescence intensity of 2'-7'-dichloroflorescein (DCF).^14^At the indicated time post HY-SDT, the cells were incubated with 1640 medium containing 20 *μ*M DCFH-DA for 30 min at 37 °C in the dark. After rinsing, the fluorescent signals of dichloroflorescein labeling ROS formation were immediately measured via fluorescence microscope.

### Cholesterol efflux assay

The capacity of macrophage degenerating cholesterol was measured using Cholesterol Efflux Fluorometric Assay Kit (BioVision, Inc., USA) according to the manufacturer's instructions. Briefly, macrophages in 96-well plate (black plate) were firstly incubated with fluorescence-labeled cholesterol for 16 h at 37 °C. After loading with HY, macrophages were treated with cholesterol acceptors high density lipids (HDL; 50 *μ*g/well) alone or for SDT. After different treatments, the supernatant and cell monolayer solubilized by cell lysis buffer were transferred to a 96-well plate (white plate) and measured at 482/515 nm. The cholesterol efflux is calculated as follows: Cholesterol efflux%=Fluorescence intensity of the media/(Fluorescence intensity of the cell lysate+media) × 100.

### DIL-ox-LDL binding assay

DIL-ox-LDL (Yiyuan Biotech, Guangzhou, China) was used to track the uptake of ox-LDL. After different treatments post HY-SDT, macrophages were incubated with DIL-ox-LDL (50 *μ*g/ml) for 6 h at 37 °C in the dark and then measured by fluorescence microscope at 488/565 nm.

### Oil red O staining

To insure the success on the inhibition of lipid accumulation and foam cell formation by HY-SDT, we used ORO (Sigma, St. Louis, MO, USA) staining. The differentiated macrophages at 80% confluence loaded with ox-LDL (50 *μ*g/ml, Yiyuan biotech, Guangzhou, China) in complete medium for 72 h were fixed with 4% paraformaldehyde, followed by dehydration with 60% isopropanol for 2 min. Next, the neutral lipids were stained using filtered 0.3% ORO solution for 10 min at room temperature. Subsequently, hematoxylin was used to counterstain the cell nuclei for 1 min after rinsing with 60% isopropanol and ddH_2_O. The ORO-stained LDs were observed by light microscope. To quantify lipid accumulation, ORO was eluted with 100% isopropanol and the optical densities of the eluates were measured using a spectrophotometer at 520 nm.

### Western blot analysis

After treatments, the cells were lysed in RIPA buffer containing protease and phosphatase inhibitors (Roche) on ice. The nuclear and cytosolic fractions were obtained by nuclear/cytosol fractionation kit (Beyotime, Beijing, China). After quantification and denaturation, equal amounts of protein samples were electrophoresed in SDS-polyacrylamide gel and transferred onto PVDF membranes (Millipore, Schwalbach, Germany), followed by blocking for 2 h at room temperature with 5% dried skimmed milk in Tris-buffered saline with 0.05% Tween 20. The membranes were probed with specific primary antibodies at 4 °C overnight with slight agitation and then incubated with Horseradish peroxidase (HRP)-conjugated secondary antibodies for 2 h at room temperature. Immunoreactivity were visualized by chemiluminescence method using ChemiDoc^TM^ MP Imaging System (Universal Hood III, Bio-Rad Laboratories, Inc., USA). The bands of proteins on the blots were quantified using Quantity One software analysis (Bio-Rad Laboratories, Hercules, CA, USA) and normalized to *β*-actin or PCNA.

### Quantitative real-time PCR (qRT-PCR)

Total RNAs were extracted using Trizol reagent (Invitrogen, Carlsbad, California, USA) and reverse-transcribed using the RT Easy II First Strand cDNA Synthesis Kit (FORGENE, Sichuan, China). Then, 1 *μ*l of cDNA was amplified in a Real-Time PCR Easy (SYBR Green I) (FORGENE, Sichuan, China) on ABI 7900HT Sequence Detection system (ABI Applied Biosystems, Foster City, CA). The following primers were used: ABCA1, forward 5′-TGAAAAAGGAGGACAGTGTTTCT-3′ and reverse, 5′-GCAGCTTCATATGGCAGCAC-3′ ABCG1, forward 5′-TCTTCGTCAGCTTCGACACCA-3′ and reverse 5′-TCTCGTCGATGTCACAGTGCAG-3′ SR-B1, forward 5′-ATGAAATCTGTCGCAGGCATTG-3′ and reverse 5′-TGCATCACCTTGGGCATCA-3′ CD36, forward 5′-AGGACTTTCCTGCAGAATACCA-3′ and reverse 5′-ACAAGCTCTGGTTCTTATTCACA-3′ SR-A, forward 5′-CCGGAAGGCCAGGAAATTCT-3′ and reverse 5′-AAGAGGGCCCTGCCCTAATA-3′. Gene expression values were normalized against that of GAPDH. Fold induction was calculated using the levels of expression of each gene at time 0 (uninfected) in THP-1-Vector cells as a reference.

### Statistical analysis

All data are presented as the mean±S.D. (at least three independent experiments). Differences among the treatment groups were assessed with one-way analysis of variance. Statistical significance was defined as *P*-value <0.05.
