# Author Correction: Hypericin-mediated sonodynamic therapy induces autophagy and decreases lipids in THP-1 macrophage by promoting ROS-dependent nuclear translocation of TFEB

**DOI:** 10.1038/s41419-019-1321-y

**Published:** 2019-02-27

**Authors:** Xuesong Li, Xin Zhang, Longbin Zheng, Jiayuan Kou, Zhaoyu Zhong, Yueqing Jiang, Wei Wang, Zengxiang Dong, Zhongni Liu, Xiaobo Han, Jing Li, Ye Tian, Yajun Zhao, Liming Yang

**Affiliations:** 1 Department of Pathophysiology, Key Laboratory of Cardiovascular Pathophysiology, Harbin Medical University, Harbin, China; 2 Department of Respiratory Medicine, The Fourth Affiliated Hospital of Harbin Medical University, Harbin, China; 3 Department of Cardiology, The First Affiliated Hospital, Cardiovascular Institute, Harbin Medical University, Harbin, China; 4 Department of Electron Microscopic Center, Basic Medical Science College, Harbin Medical University, Harbin, China; 5 Division of Cardiology, The First Affiliated Hospital, Harbin Medical University, Harbin, China

**Correction to:** **Cell Death and Disease** (2016) **7**: e2527

10.1038/cddis.2016.433

published online 22 December 2016

The authors wish to point out that in Fig. 1f, the picture of DAPI in the ATG5 siRNA group is incorrect. During the process of image synthesis, the authors mixed the pictures of DAPI in the control group and ATG5 siRNA group, leading to the duplicate between them of DAPI. Furthermore, the AMPK blot and the AKT blot in Fig. 2a were inadvertently duplicated with the third β-actin in Fig. 2a and AKT in Fig. 4e, respectively. The authors would like to apologize for any inconvenience this may have caused.

The correct figures are presented below.

**Fig. 1:**
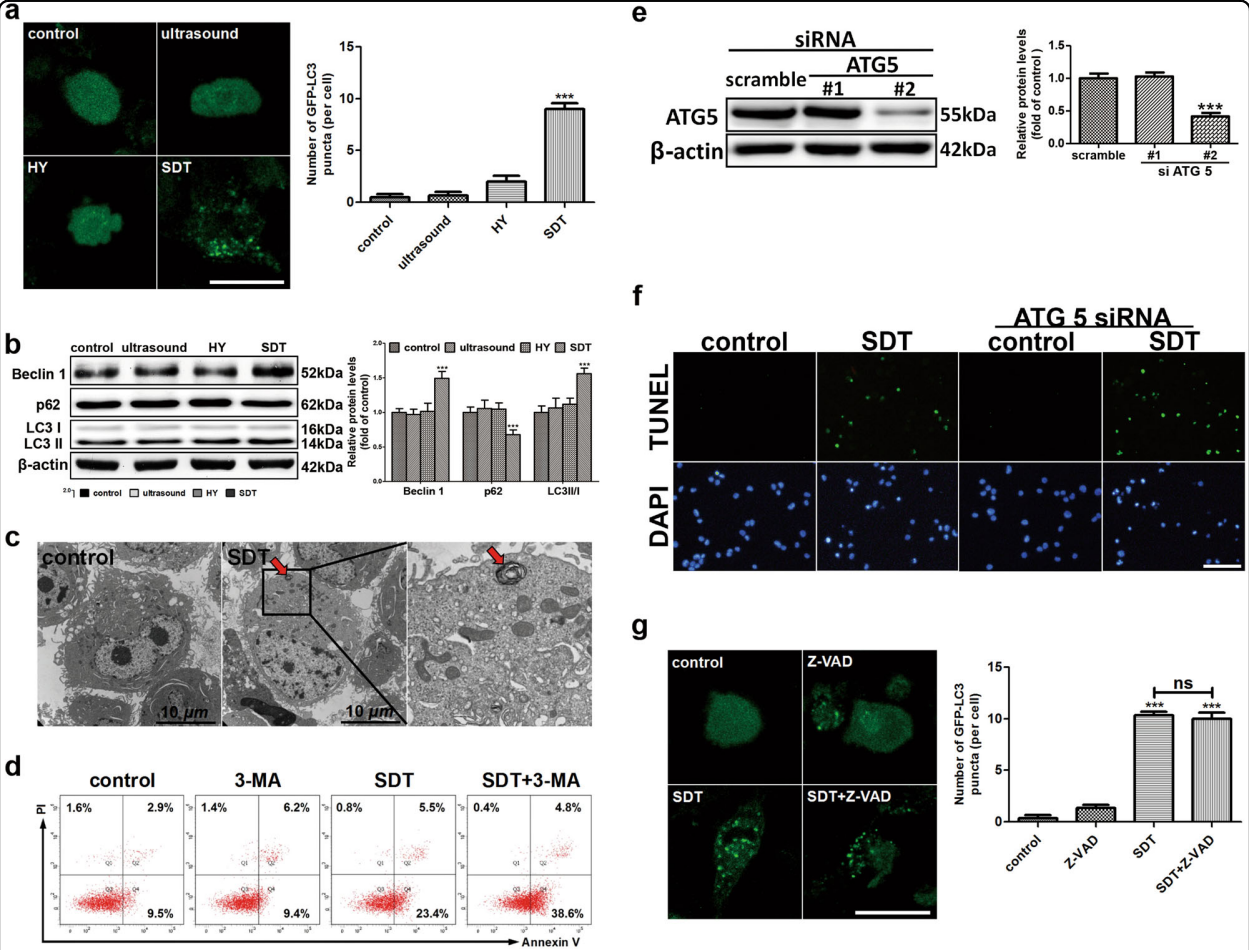
▓

**Fig. 2:**
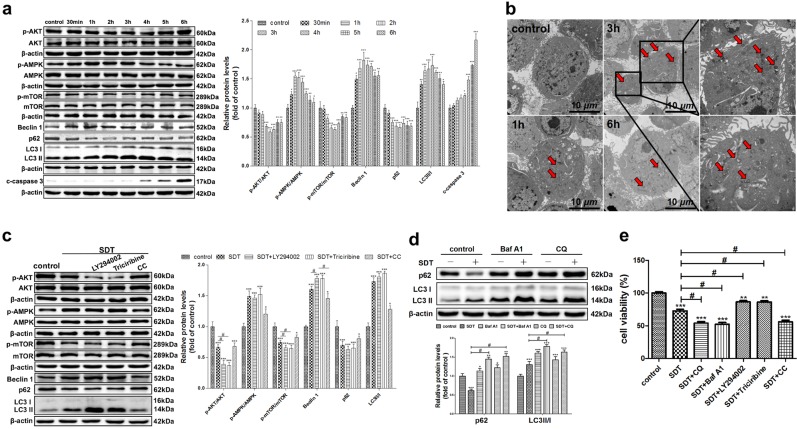
▓

