# Supplementary figures and images for: Hypericin-mediated sonodynamic therapy induces autophagy and decreases lipids in THP-1 macrophage by promoting ROS-dependent nuclear translocation of TFEB
Source: Cell Death Dis. 2016 Dec 22;7(12):e2527–. doi: 10.1038/cddis.2016.433 (PMC5260986; doi:10.1038/cddis.2016.433)

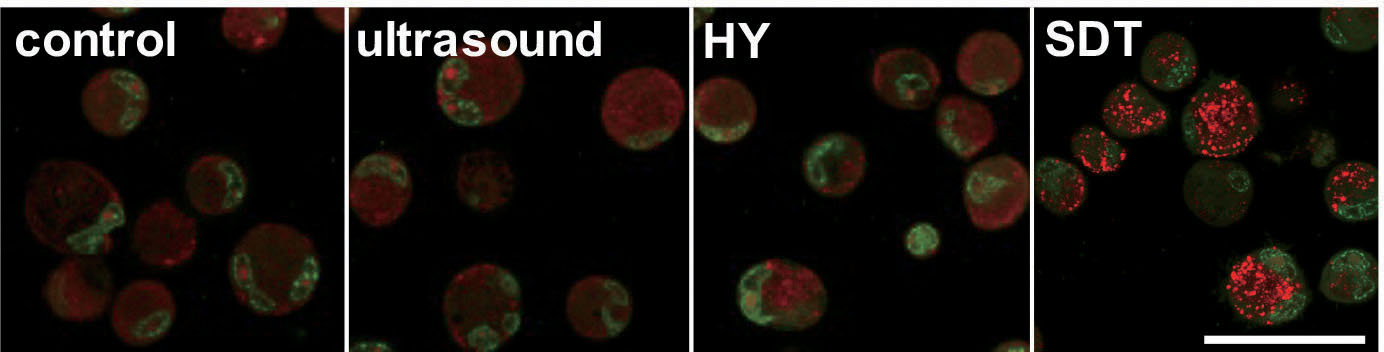

Supplement: Supplementary Figure 1 [file cddis2016433x1.tif]

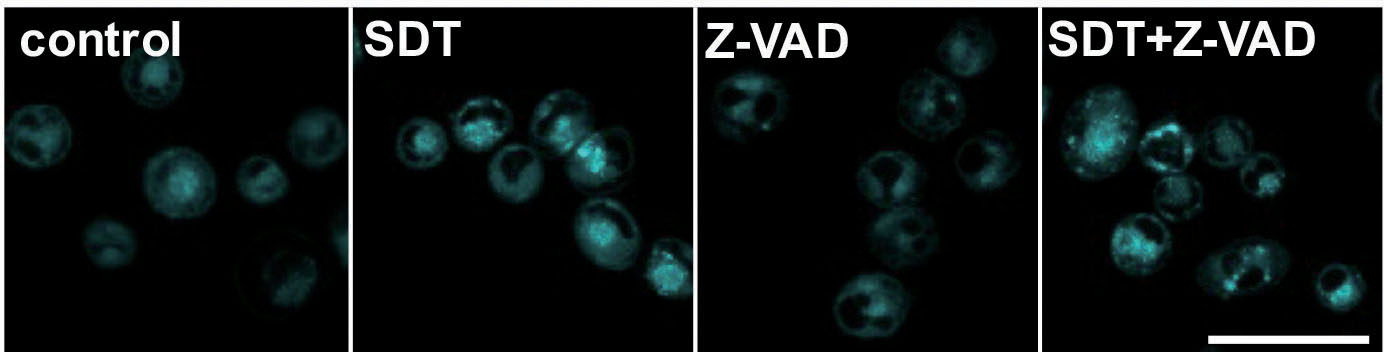

Supplement: Supplementary Figure 2 [file cddis2016433x2.tif]

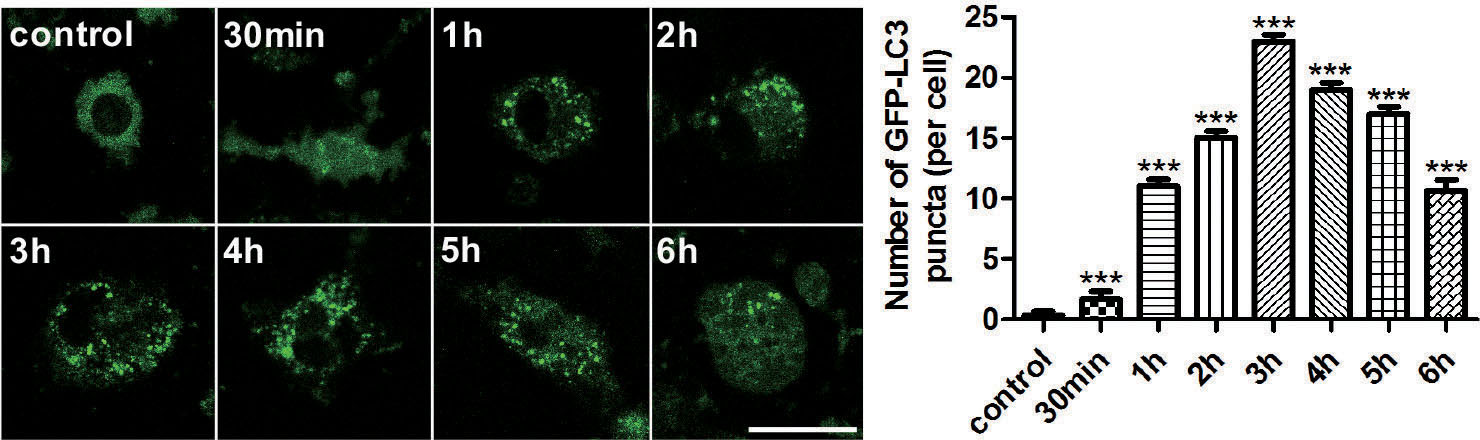

Supplement: Supplementary Figure 3 [file cddis2016433x3.tif]

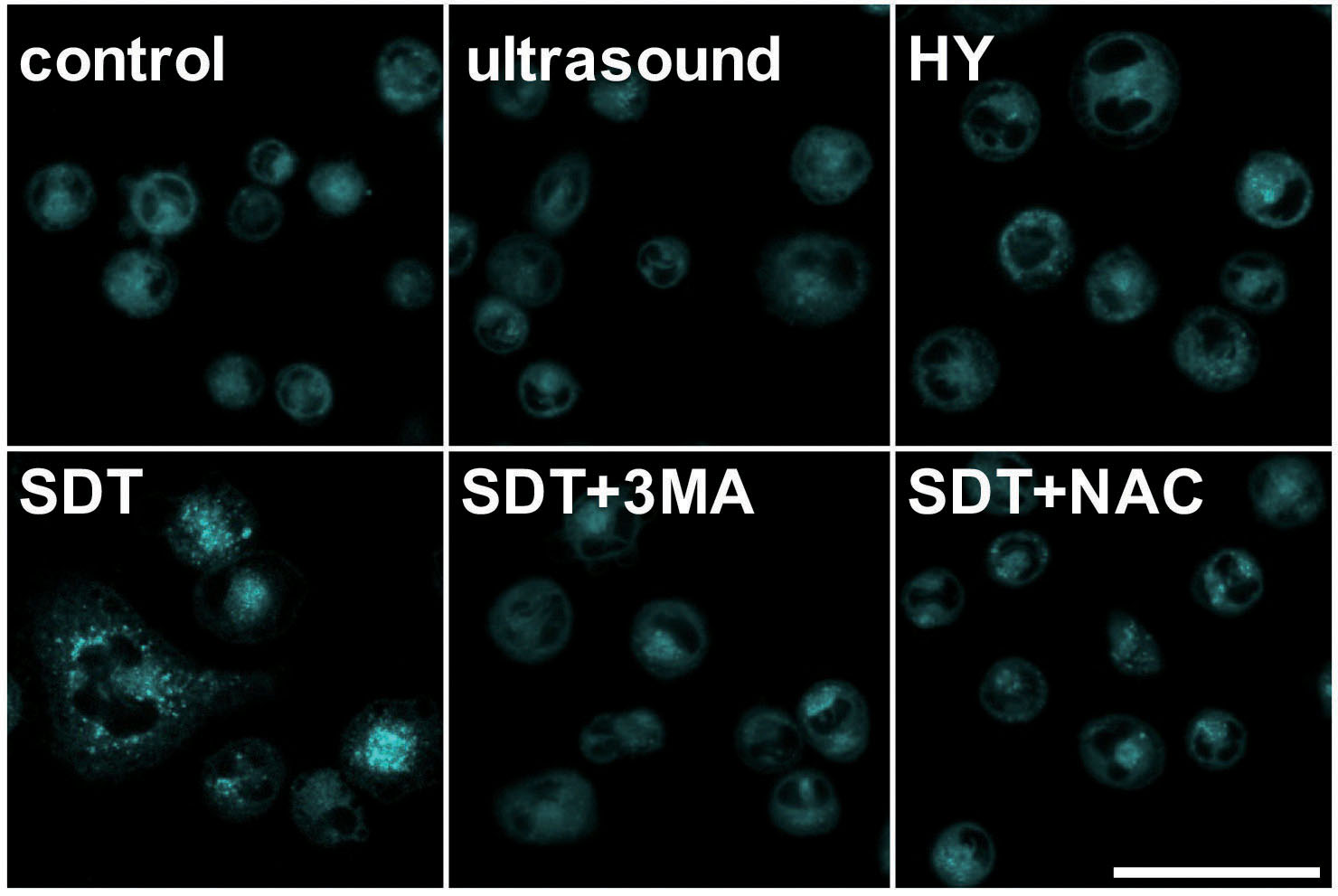

Supplement: Supplementary Figure 4 [file cddis2016433x4.tif]

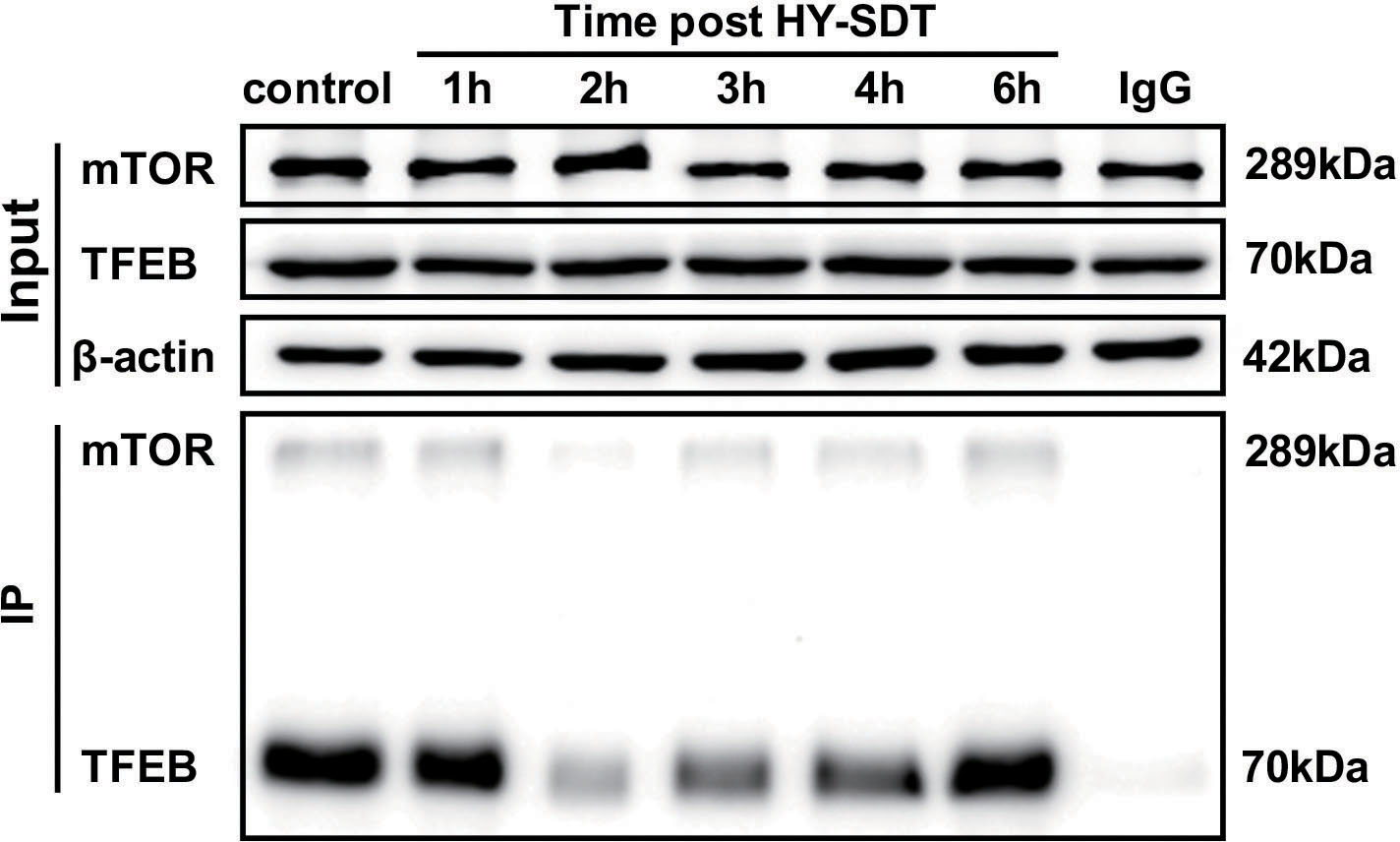

Supplement: Supplementary Figure 5 [file cddis2016433x5.tif]

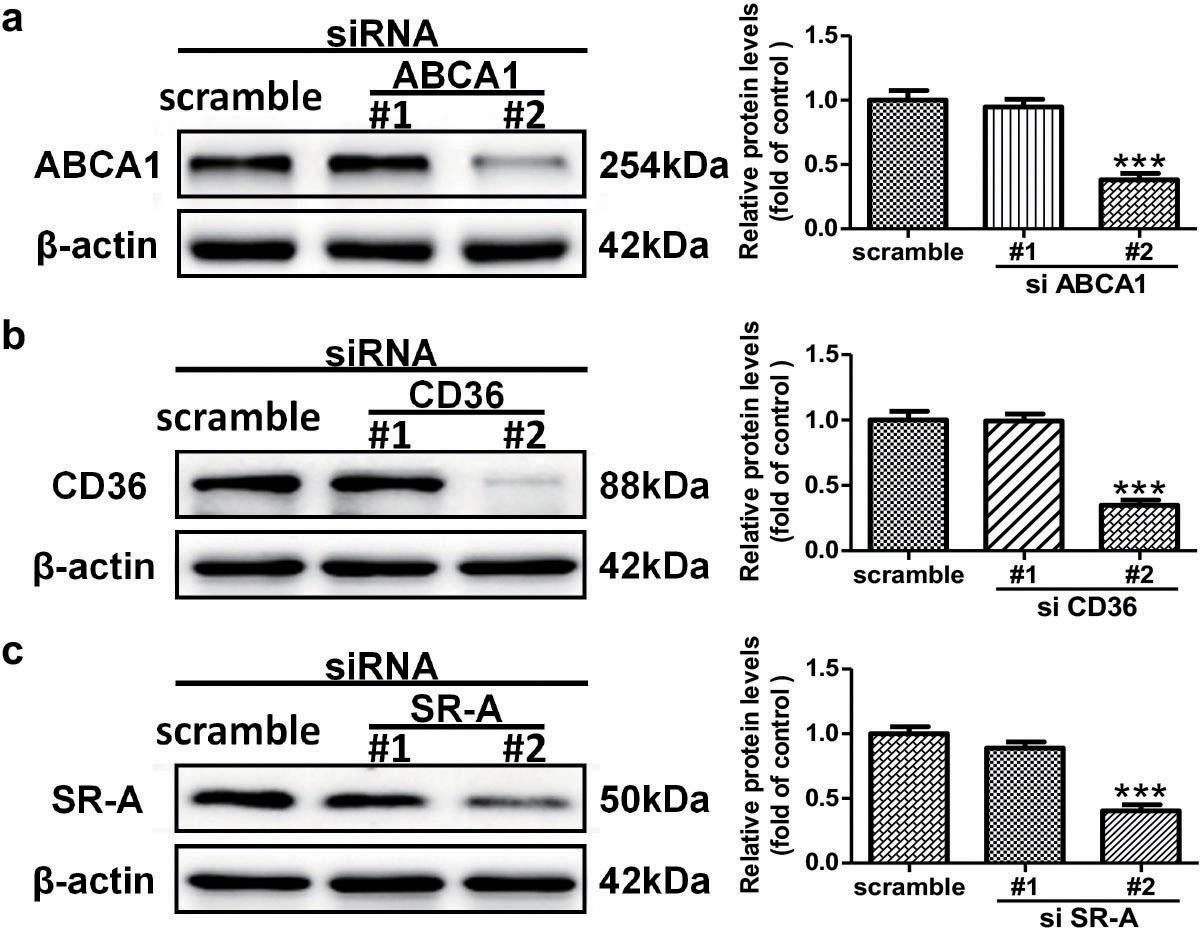

Supplement: Supplementary Figure 6 [file cddis2016433x6.tif]
