# Supplementary material for: Hypericin-mediated sonodynamic therapy induces autophagy and decreases lipids in THP-1 macrophage by promoting ROS-dependent nuclear translocation of TFEB
Source: Cell Death Dis. 2016 Dec 22;7(12):e2527–. doi: 10.1038/cddis.2016.433 (PMC5260986; doi:10.1038/cddis.2016.433)
Supplement: Supplementary Information [file cddis2016433x7.docx]

**Hypericin-mediated sonodynamic therapy** **induces autophagy and decreases lipids in THP-1 macrophage by promoting ROS-dependent nuclear translocation of TFEB**

Running title: Autophagy induction and lipid reduce in macrophage

X Li^1,6^, X Zhang^2,6^, L Zheng^1^, J Kou^1^, Z Zhong^1^, Y Jiang^1^, W Wang^4^, Z Dong^4^, Z Liu^1^, X Han^1^, J Li^5^, Y Tian^*,1,3^, Y Zhao^*,1^, L Yang^*,1^

**Supplementary Methods**

**Acridine organe (AO) and monodansylcadaverine (MDC) staining.** AO is sensitive to acidic vesicular organelles (AVOs), such as lysosome and autolysosome, and used to detect autophagy. It can stain DNA and cytoplasm bright green (AO^-^), while in the presence of acid compartments it fluoresces bright red (AO^+^). To assess changes in the formation of intracellular AVOs, at 2 h or 6 h following HY-SDT, cells were stained with 1 *μ*g/ml AO according to published procedures^36^ and measured by confocal laser scanning microscope (CLSM) (LSM 510 Meta; Zeiss, Gottingen, Germany) and quantified with flow cytometry. MDC is a widely used special tracer for autophagic vacuoles. To explore the impact of ROS and apoptosis on autophagic vacuoles formation, the cells stained with 20 mM MDC were detected under CLSM. Cells having fluorescent dots or vacuoles were considered MDC positive.

**Co-Immunoprecipitation (CO-IP).** The total proteins were extracted using specific cell lysis buffer (Beyotime, Beijing, China). The extracts containing 1 mg protein were incubated with anti-TFEB and IgG (1:100 v/v) overnight at 4 °C on a rotary shaker. Then 20 *μ*l Protein G agarose beads (Santa Cruz, CA, USA) were added and the mixture was incubated for 1 h at 4 °C. After washing with cell lysis buffer, the immunoprecipitate resuspended in 4X sample buffer and separated on 10% SDS-polyacrylamide gel. The following protocol was as the same as the method of western blot.

**Figure Legends**

**Supplementary Figure S1** Autophagy activation of macrophage following HY-SDT. Detection of macrophage autophagy following HY-SDT by AO staining under a CLSM. The number of AO^+^ cells significantly increased in the HY-SDT group. Scare bar=50 *μ*m.

**Supplementary Figure S2** The effect of Z-VAD on the autophagy activation of macrophage following HY-SDT. Detection of macrophage autophagy with or without Z-VAD following HY-SDT by MDC staining under a CLSM. Scare bar=50 *μ*m.

**Supplementary Figure S3** Detection of autophagy in GFP-LC3 transfected macrophage at different time points following HY-SDT examined under a CLSM.

Scare bar=20 *μ*m. ****P* < 0.001 vs control. All values are given as mean ± S.D. (error bars) of three independent experiments

**Supplementary Figure S4** The effects of autophagy inhibitor 3-MA and ROS scavenger NAC on autophagy activation of macrophage following HY-SDT. The cells with different treatments were examined under a CLSM by MDC staining. Bar=50 *μ*m.

**Supplementary Figure S5** Detection of interactions between TFEB and mTOR at different time points following HY-SDT by CO-IP.

**Supplementary Figure S6** The expression levels of ABCA1, CD36 and SR-A following siRNA treatments by western blot. (a) Representative western blots of ABCA1 following siRNA treatment. (b) Representative western blots of CD36 following siRNA treatment. (c) Representative western blots of SR-A following siRNA treatment. ****P* < 0.001 vs control. All values are given as mean ± S.D. (error bars) of three independent experiments
